# Supplementary material for: The MyoD1 Promoted Muscle Differentiation and Generation by Activating CCND2 in Guanling Cattle
Source: Animals (Basel). 2022 Sep 26;12(19):2571. doi: 10.3390/ani12192571 (PMC9559206; doi:10.3390/ani12192571)
Supplement: Supplementary file 1 [file animals-12-02571-s001.zip › animals-1832941-supplementary.pdf]

animals

MDPI

Supplementary

1. MyoD1 gene knockouts effect

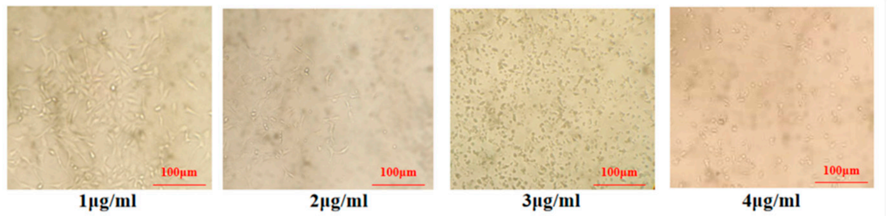

Figure S1. MDBK cell survival in the presence of the indicated concentrations of puromycin after 48 h.

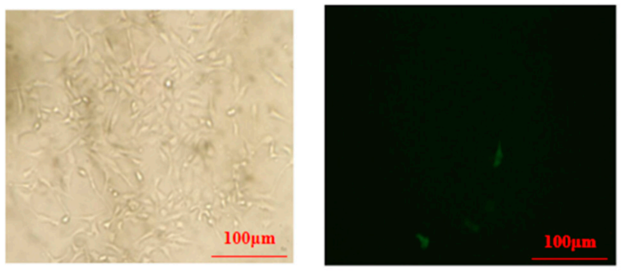

Figure S2. Lipofectamine transfection of GFP control plasmid pX458. Right panel, light micrograph of transfected cells; Left panel, fluorescent microscopy using phase contrast.

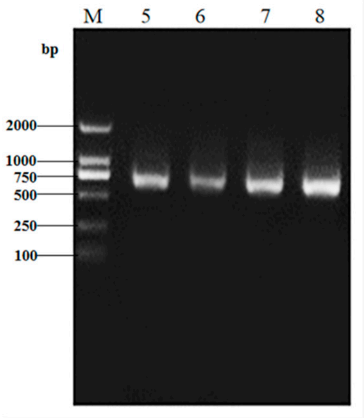

Figure S3. PCR amplification of genomic DNA taken from 4 CRISPR targets in MDBK cells. 5,6,7,8: Respectively represent the gel band size of g5,g6, g7 and g8.

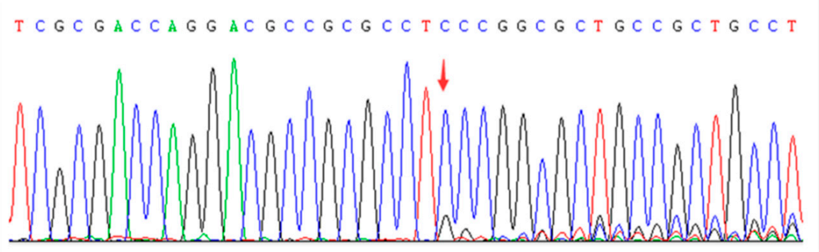

Figure S4. Analysis of gRNA target sequences.

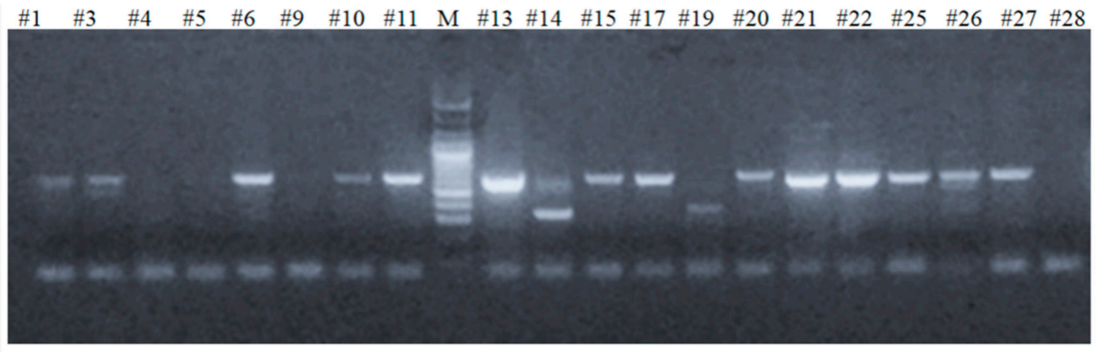

Figure S5. Gel electorophoresis of PCR amplicons generated from *MyoD1* gene disruptions in cell lines identified using limiting dilution. M, DNA marker(Takara, Dalian, China).

2. GO Functional annotation of DEGs

Table S1. GO enrichment analysis of DEGs.

**GO ontology**		**Cluster Frequency**	**Genome Frequency of Use**	**Corrected *p*-Value**
**Cellular component**	Plasma membrane	19.6%	10.7%	7.51 E-10
	extracellular matrix	5.4%	1.6%	8.77 E-8
	intrinsic component of membrane	38.0%	30.6%	0.00694
	Membrane	51.0%	43.4%	0.01055
	Extracellular region part	13.0%	7.2%	0.0000166
**Molecular function**	integrin binding	2.4%	0.5%	0.0000431
	Signaling receptor binding	11.7%	6.8%	0.00092
	Cell adhesion molecule binding	3.1%	0.9%	0.00063
	Calcium ion binding	6.3%	3.3%	0.02028
	transmembrane transporter activity	8.7%	5.4%	0.10275
**Biological process**	cell differentiation	19.9%	13.9%	0.02449
	striated muscle tissue development	2.5%	1.3%	1
	muscle structure development	3.7%	1.9%	1
	muscle cell differentiation	2.1%	1.0%	1
	muscle organ development	2.4%	1.1%	1
